# Environmental influences on community participation among people with multiple sclerosis: A mixed methods study

**DOI:** 10.1371/journal.pone.0342678

**Published:** 2026-02-10

**Authors:** Bishan Yang, Ivan Molton, Andrew Humbert, Carolyn Baylor, Emma Gregg, Dawn Ehde, Jennifer Sullivan, Elysa Lanz, Michael Schiller, Philip Hurvitz, Danbi Lee

**Affiliations:** 1 Department of Rehabilitation Medicine, University of Washington School of Medicine, Seattle, Washington, United States of America; 2 National Multiple Sclerosis Society, the Greater Northwest Chapter, Seattle, Washington, United States of America; 3 Community advisory board member, Seattle, Washington, United States of America; 4 Center for Studies in Demography and Ecology, University of Washington, Seattle, Washington, United States of America; University of Illinois Urbana-Champaign, UNITED STATES OF AMERICA

## Abstract

**Objective:**

To examine the influence of environmental factors (EFs) and personal factors (PFs) on community participation among people with multiple sclerosis (PwMS) and identify areas for improvement.

**Methods:**

A mixed methods explanatory sequential design was used. A secondary data analysis of patient-reported outcomes and Global Positioning System (GPS) data was completed using multiple linear regression analysis to examine associations between five EFs, five PFs, and six community participation outcomes in 100 PwMS. Four focus groups were completed with 12 PwMS who use mobility aids and 12 who do not to understand how EFs affected community participation experiences. Thematic analysis was used.

**Results:**

Regression results showed significant associations between PFs and five community participation outcomes (R^2^ = 13% − 48%, p < 0.05), and EFs explained an additional 11% variation in satisfaction with participation and 11% in GPS-derived measures of activity space, after adjusting for PFs (ΔR² = 0.11, p < 0.05). Among individual EFs, after accounting for PFs, perceived financial resources was associated with ability to participate (B = 1.46, p = 0.018), and satisfaction with participation (B = 3.12, p < 0.001). Social support (B = −1.05, p = 0.022) and neighborhood safety (B = 1.3, p = 0.007) were associated with activity space. Qualitative findings revealed that mobility aid users experienced increased challenges in the built environment, and non-users reported more concerns about the attitudinal environment. They also described how environmental support enabled participation despite functional declines. Acceptance and adaptation were useful strategies, but participants called for improvements in the built environment, information access, MS specialty care, and public attitudes towards disability.

**Conclusion:**

Community participation among PwMS is influenced by both PFs and EFs. Statistically, EFs uniquely affected participation satisfaction and activity space, while qualitative findings revealed major barriers and highlighted needs for improvement in physical, social, and attitudinal environments.

## Introduction

Multiple Sclerosis (MS) is an inflammatory and neurodegenerative disease affecting approximately 2.2 million adults worldwide, including one million in the US [1,2]. MS is characterized by a range of visible symptoms (e.g., mobility impairments) and invisible symptoms, such as fatigue, pain, cognitive problems, sensory issues, and psychological disorders [3]. The disease is currently divided into four types—clinically isolated syndrome, relapsing-remitting, secondary progressive, and primary progressive— with the initial diagnosis typically occurring around age 30 [4]. Although various disease-modifying therapies are available and are effective in reducing relapse frequency and delaying progression, the cause of MS remains unknown, and there is not a cure. Experiences living with MS are highly individualized, but many people with MS (PwMS) face critical challenges in maintaining sufficient and meaningful participation in life activities [5,6]. Participation challenges intensify in out-of-home community contexts (e.g., workplaces, public spaces) where environmental conditions are less controllable [7–9].

Community participation is an important health outcome significantly linked to quality of life among PwMS [10,11]. Factors contributing to community participation among those living with chronic neurological conditions are multifaceted [12,13]. These factors include not only personal factors (e.g., sociodemographic characteristics and functional impairments) but also external factors in the physical, social, and attitudinal environments, such as architectural accessibility, socialization opportunities, and public attitudes toward disability. The significant impact of personal factors (PFs), such as age, disease duration, physical function, fatigue, depression, anxiety, and pain, on participation of PwMS has been well-supported in a large body of systematic reviews and empirical studies [6,14–20]. Based on this rich research evidence, participation interventions for the MS population have primarily focused on improving physical and/or psychological function [18,21].

Environmental influences on community participation can be complex and associated with systemic structures and policies. Prior MS studies have investigated both PFs and environmental factors (EFs) and highlighted the critical roles of EFs in participation. For example, in quantitative literature, Plow et al. [22] identified environmental barriers as a significant predictor of community participation, along with physical and cognitive impairments and self-management skills. Lehmann et al. [23] reported that job resources and demands were more influential for job retention than PFs such as age, MS duration, and symptom severity. Several qualitative studies revealed that PwMS attributed limited community participation mostly to extrinsic barriers, such as limited accessibility in the built environment, lack of access to services and accommodations, and judgmental attitudinal environments [24–26]. A mixed methods study showed that PFs explained 20% of the variance in work participation, while qualitative data attested to the importance of EFs (e.g., job flexibility, family support) [27].

Despite emerging evidence of the importance of EFs in community participation, current MS interventions primarily focus on managing the effects of symptoms and improving physical function using strategies such as medication, pacing, meditation, and exercise to facilitate participation [21,28]. Interventions focusing on empowering PwMS to navigate environmental barriers and advocating systemic support are limited [29]. Calls for system-level change are relatively rare in the MS literature. Further research is needed to comprehensively assess the effects of EFs on community participation among PwMS and identify desired systemic changes to promote community participation.

Conceptual models, such as the 1997 Institute of Medicine Model of disability [30] and Lawton’s Environmental Press Theory [31], have illustrated the dynamic person-environment interaction in shaping participation. These frameworks suggest that EFs may modify the association between PFs and participation outcomes. For example, while environmental barriers (e.g., inaccessible architecture, discrimination) can restrict participation for a person with mobility impairments, supportive environments, such as a wheelchair or positive social support, should mitigate the negative impact of mobility difficulties and facilitate participation. Although the person-environment-participation relationships have been described in people with disability due to other neurological conditions (e.g., spinal cord injury [32]), less work has addressed the interaction of these factors in PwMS. A better understanding of these interactions could advance the development and application of individual rehabilitation interventions addressing many of the issues that PwMS see as barring their access to community participation. Such knowledge may also inform public policy addressing societal barriers to participation in this population.

This study addresses existing research gaps through a mixed methods approach with three aims: (1) To examine the influence of EFs on community participation among PwMS, using quantitative analyses to assess direct associations and potential moderation effects with PFs. (2) To explore how PwMS perceive the role of EFs in shaping their community participation experiences and identify their priorities for systemic support through qualitative inquiry, and (3) To integrate quantitative and qualitative findings to generate a more comprehensive understanding of how EFs impact community participation and inform system-level recommendations.

## Materials and methods

### Study design

This mixed methods study employed an explanatory sequential design [33], beginning with a quantitative phase followed by a qualitative phase. The quantitative phase was a secondary analysis assessing the relationship between PFs, EFs, and community participation outcomes, using deidentified baseline data from an existing clinical trial conducted in the U.S. (R01NR016942, Molton PI, University of Washington IRB #00003931, access to deidentified dataset granted for the first author in June 2023). In the second phase, we conducted online focus groups to explore how EFs affected out-of-home participation, strategies used to manage barriers, and desired system-level changes among PwMS. A community advisory board (CAB) comprising three individuals (co-authors, JS, EL, MS) with expertise and lived experience contributed to focus group planning, co-facilitation, and result interpretation throughout the study. This mixed methods study was reviewed and approved by the University of Washington Human Subjects Division (IRB ID: STUDY0018560) with an exempt status, and the need for formal consent was waived. This manuscript follows the American Psychological Association Mixed Methods Journal Article Reporting Standards [34].

## Quantitative phase

### Data source and sample

Quantitative data were drawn from a clinical trial evaluating a telehealth-based health promotion program for people with physical disabilities [35]. Participants were U.S. adults aged 45–64 who had acquired physical disability prior to age 40. Baseline (pre-randomization) data were collected from PwMS between April 2019 – September 2021, and were used in the present analysis. All participants completed a battery of self-report outcomes, including measures of health and participation. A randomly selected 50% of participants were then also invited to join a Global Positioning System (GPS) sub-trial, where they were asked to wear a QStartz BT Q1000XT GPS logger for a continuous seven-day period to monitor real-time community mobility. A total of 122 participants with MS were invited to participate in the GPS sub-trial, and of those, 100 individuals consented and provided valid survey and GPS data, comprising the final quantitative sample for this study.

### Outcome variables: Community participation measures

Community participation outside the home can be divided into subjective aspects that are known only to the individual (e.g., satisfaction) and objective aspects reflecting the quantity of engagement, such as frequency of activity engagement and activity space [36,37]. The importance of measuring both the objective and subjective aspects to fully capture experiences has been emphasized in literature [38]. While self-reported methods are valid and reliable for subjective aspects of participation (e.g., satisfaction), real-time measures, such as GPS data, have been recommended for objective aspects of community participation to improve data accuracy [36,39]. In this study, we included both participant-reported outcomes of community participation and GPS-measured community mobility performance variables.

Subjective participation measures included the self-reported PROMIS Ability to Participate in Social Roles and Activities (PROMIS-AP) and PROMIS Satisfaction with Social Roles and Activities short forms (PROMIS-SRA) [40]. A T-score metric was used to interpret PROMIS scores. While in all PROMIS scales, a T-score of 50 (SD = 10) represents the general population mean, domain-specific cut points were referenced to interpret impairment severity [41]. A PROMIS-AP score above 45 suggests a level of functioning within the national normal range, and PROMIS-SRA scores between 40–60 reflect an average level of satisfaction [41]. In both cases, higher T-scores correspond to more favorable participation outcomes.

Community mobility was quantified through four GPS-derived indicators. Three were related to the concept of “trips,” defined as travel episodes that started and ended at the person’s residence and included any stops in between. These variables were: 1) daily trip count (trip frequency), 2) total kilometers traveled per day (trip distance), and 3) total hours spent on trips (trip duration). The fourth metric, radius of gyration (in kilometers), is an indicator of an individual’s activity space. It is calculated as the root mean square of the distances from each GPS point to the centroid of all GPS points (i.e., home location), representing the geographic spread of the individual’s travel throughout the monitoring period [42]. Higher values indicate that participants traveled over a wider area, regardless of the specific activities they engaged in. Methods used to process GPS data to identify trip-related variables have been described elsewhere (Yang et al., 2025) [43]  and are available with the authors upon request. GPS data were managed and analyzed using PostgreSQL v. 14.18 (https://www.postgresql.org/) and PostGIS v. 3.5 (http://postgis.net/).

### Independent variables: Personal factors and environmental factors

PFs were comprised of demographic information and MS-related symptoms, all collected during the baseline survey. Demographic data included age, disease duration, self-reported sex, race/ethnicity, level of education, income, marital status, employment, and comorbidity. MS-related symptom variables included physical function, fatigue, and depression. These were assessed using standardized PROMIS short forms [44–46]. PROMIS T-scores above 45 on the physical function scale indicate typical physical functioning, while scores below 55 on the fatigue and depression scales reflect normative levels [41]. Higher T-scores indicate more of the construct being measured—greater physical ability on the physical function scale and greater symptom severity on the fatigue and depression scales.

EFs were also captured using the baseline survey. Variables included in this analysis were perceived social support, perceived financial resources, and perceived neighborhood safety, measured by three single items from the Older People’s Quality of Life questionnaire – brief version [47]. Response options for each item range from 1–5 (1 = Strongly disagree, 2 = Disagree, 3= Neither agree or disagree, 4 = Agree, 5 = Strongly agree), with higher scores indicating greater perceived environmental support. Use of mobility aids (i.e., crutches, cane(s), walker, medical shoes, manual wheelchair, power wheelchair, scooter, brace, hiking poles) was also included as an EF. Those who reported use of a mobility aid were further categorized into two groups: (a) using walking aids only, (b) using wheeled aids, inclusive of those using both wheeled aids and walking aids.

### Quantitative data analysis

Quantitative analyses were conducted using IBM SPSS Statistics (Version 27). Descriptive analysis was conducted for all variables to report mean, percentage, and standard deviation (SD). We assessed the association between EFs and community participation in two ways. First, multiple linear regression with a stepwise entry was used to examine the unique association between EFs and participation outcomes after controlling for PFs, as a block. PFs (i.e., age, disease duration, physical function, fatigue, depression) were entered in Block 1, and EF variables (i.e., perceived social support, perceived financial resources, and perceived neighborhood safety) were then entered into Block 2. We observed whether the addition of EF variables resulted in an additional explanation of variance in the outcome variable, indicated by a significant ΔR^2^. Six regression models were established, two for participant-reported participation outcomes (PROMIS-AP and PROMIS-SRA) and four for GPS-derived participation outcomes (trip frequency, trip distance, trip duration, and radius of gyration).

Second, we evaluated potential moderating effects of specific EFs on associations among PFs and community participation outcomes, using multiple linear regression. Based on existing literature [48,49] and to manage risk of family-wise error, we focused on evaluating three specific potential moderating relationships: (1) whether the use of mobility aids moderated the association between physical function and community participation; (2) whether social support moderated the association between fatigue severity and community participation; and (3) whether social support moderated the association between depression severity and community participation. Each moderation was tested for the listed six participation outcomes, and included both main effects for the EF and PF of interest, and their interaction, after adjusting for age and disease duration.

Guided by prior research and theoretical considerations, we selected independent variables in the regression models *a priori* rather than through correlation screening. We assessed the assumptions of multiple linear regression and found no violations of independence, linearity, homoscedasticity, or multicollinearity. However, three variables (trip distance, trip duration, and radius of gyration) required natural log transformation to ensure normality for regression analysis. For all aforementioned analyses, associated p-values <0.05 (two-tailed tests) indicate statistically significant results. In the absence of multiple comparison correction, all quantitative results are exploratory and should be interpreted with caution.

## Qualitative phase

### Online focus group recruitment

Upon the completion of the quantitative analysis, convenience and purposive sampling methods were used to recruit participants across the U.S. for online focus groups from November 2023 to January 2024. A screening survey was distributed through the University of Washington MS Rehabilitation and Wellness Center national registry and the National MS Society website and their registry for three states representing the West, Midwest, and Northeast regions, respectively. The survey gathered information on demographics, mobility aid use, and interest in study participation. Eligibility criteria included being an adult with MS, having access to Zoom, the ability to communicate effectively in English, and a willingness to participate in the focus groups. Participants were purposively selected and invited to ensure balanced mobility aid users and nonusers and diverse backgrounds of gender, race, MS duration, location, and employment status. Of note, we did not directly reach out to participants of the first phase or ask whether they had participated in the parent study, therefore, focus group participants may or may not have participated in the first phase.

### Focus group data collection

Focus groups were held via Zoom. Based on the CAB’s suggestions and previous literature showing that participation experiences differ significantly between PwMS who use mobility aids versus those who do not [25], participants were grouped based on their mobility aid use status (hereafter referred to as “mobility aid users” or “users” versus “nonusers” or “those living with invisible MS”). A discussion guide was developed with insights from quantitative findings and the CAB’s input to prompt conversations about how PFs and EFs affect daily community participation experiences. An additional question inquiring about the experience of using mobility devices in the community was asked in focus groups with mobility aid users. Table 1 presents example questions. To orient participants, discussion points were shared with them one day before the scheduled focus group. Each focus group was facilitated by the lead researcher and at least one CAB member. All online focus group discussions were recorded on Zoom, and audio recordings were transcribed verbatim. Prior to recording, participants provided verbal consent and confirmed agreement through Zoom’s embedded consent feature.

**Table 1:** Example focus group questions.

1. Describe 1 or 2 environmental/external factors that make it most difficult for you to do things outside the home while living with MS.
2. How do you manage those most difficult situations/significant barriers in order to go out and do things as wished? What would have better supported you? What changes would you like to see or advocate for?
3. [For mobility aid users only] Some people see that using mobility aid devices in the community is a support, while others say that it’s a barrier, what has been your experience? For those who use more than one kind of device, how does your experience differ by device?

### Qualitative and integrated data analysis

Thematic analysis [50] with a combination of deductive and inductive coding [51] was used to analyze focus group data. Pre-specified code groups and codes were established by the lead researcher based on research questions and literature review. Inductive coding added data-driven codes. Two coders participated in the coding and theme development process. They independently analyzed one transcript from each group (mobility aid user and nonuser groups) to draft the initial code book, which was then refined through iterative coding of the remaining transcripts. A third researcher who was familiar with all transcripts participated in meetings to support the consensus-building process. Memos were written throughout the coding process to record coders’ impressions of each transcript and comparative insights between mobility aid users and nonusers. Final code groups and names, definitions, and related quotations were compiled in a coding report generated by ATLAS.ti. Themes and subthemes were developed using memos and the coding report, then revised and finalized by consensus among the research team. To enhance trustworthiness, member checking was conducted by emailing focus group participants a two-page summary of results and request for feedback. Eight participants provided feedback, which was used to refine the reporting of qualitative results.

As part of the explanatory sequential mixed methods design, an integrated analysis was conducted to explore how qualitative findings explained, contextualized, and contrasted with quantitative results [33]. During this process, attention was given to how qualitative findings may explain the significant statistical relationships between variables of interest or lack thereof, and how qualitative findings may contribute different insights complementary to quantitative findings. In this manuscript, integrated insights are incorporated in qualitative themes, presented in the Qualitative and Integrated Findings section.

## Results

### Participants in the quantitative phase (n = 100)

Table 2 summarizes demographic characteristics of the 100 participants in the quantitative phase. Participants had a mean age of 56 years and average time since diagnosis of 20 years. Participants resided in 29 different US states. The sample was predominantly female (80%) and non-Hispanic White (87%), consistent with known prevalence patterns of MS in the US [52]. The majority of participants were married/partnered (68%) and used a mobility aid (66%), with over half holding a college degree or higher and reporting annual household income higher than $50,000. Nearly half (n = 47) of the participants reported unemployment, mainly due to disability. Among mobility aid users, half reported using walking aids exclusively, and half used either wheeled aids only or both types. Participants rated their level of social support, financial resources, and neighborhood safety as generally high (means > 4). Compared to the US general population, participants reported below-average physical function (T-score = 41), higher fatigue severity (T-score = 60), and average depression severity (T-score = 52), based on the domain-specific cut points for PROMIS measures [41].

**Table 2:** Quantitative phase participant characteristics (n = 100).

Measure	M, T-score, or N *(SD or %)*
***Age (year)***	55.57 *(5.44)*
(Range)	(45-65)
***Disease Duration (year)***	19.65 *(8.26)*
(Range)	(1-45)
***Sex***	
Female	80 *(80%)*
Male	18 *(18%)*
Declined to answer	2 *(2%)*
***Race and Ethnicity***	
NH White	87 *(87%)*
NH Mixed-race	6 *(6%)*
NH African American	4 *(4%)*
Hispanic	3 *(3%)*
***Educational Background***	
High school graduate	3 *(3%)*
Some college	27 *(27%)*
College graduate	36 *(36%)*
Graduate school	33 *(33%)*
Declined to answer	1 *(1%)*
***Annual Household Income***	
Less than $50,000	29 *(29%)*
$50,000 to $75,000	12 *(12%)*
More than $75,000	40 *(40%)*
Declined to answer	19 *(19%)*
***Marital Status***	
Married/Partnered	68 *(68%)*
Previously partnered (divorced, separated, widowed)	20 *(20%)*
Single	10 *(10%)*
Declined to answer	2 *(2%)*
***Employment Status***	
Employed	39 *(39%)*
Unemployed due to disability	37 *(37%)*
Unemployed due to other reasons	10 *(10%)*
Retired	7 *(7%)*
On sick or other leave	2 *(2%)*
Declined to answer	5 *(5%)*
***Participation Outcomes***	
Perceived ability to participate	42.06 *(6.04)*
Satisfaction with Participation	43.13 *(7.59)*
Trip frequency (times/day)	0.94 *(0.65)*
Trip distance (km/day)	30.70 *(35.35)*
Trip duration (hour/day)	3.05 *(3.06)*
Radius of gyration (km/tracking period)	15.46 *(32.33)*
***MS-related Symptoms***	
Physical function	40.88 *(11.17)*
Fatigue severity	59.99 *(8.26)*
Depression severity	51.81 *(9.09)*
***Environmental Factors***	
Perceived social support	4.50 *(0.72)*
Perceived financial resources	4.08 *(0.92)*
Perceived neighborhood safety	4.52 *(0.69)*
Using any mobility aids	66 *(66%)*
Using walking aids only	33 *(33%)*
Using wheeled aids	33 *(33%)*

*Note.* NH = Non Hispanic. M = Mean. N = count. SD = standard deviation. “Using wheeled aids” refers to individuals who rely solely on wheeled mobility devices as well as those who use them in combination with walking aids.

Regarding community participation outcomes, participants’ satisfaction with participation (T-score = 42) was lower than the general population mean (score of 50), but still within the range of average (40–60), whereas their ability to participate (T-score = 42) indicated mild limitations [41]. Participants were tracked by the GPS device for an average of 8 consecutive days, ranging from 4–19 days, and 92% were tracked for 7 or more days. GPS data revealed that on average, participants made one trip per day, traveled for a total of 31 km and 3 hours per day. In addition, the mean radius of gyration during the entire tracking period was 15 km (SD = 33).

### Focus group participant characteristics (n = 24)

A total of 24 people from nine different states participated in four focus groups. Each group had six participants and lasted for about 90 minutes. Two focus groups were with PwMS who use mobility aids (P1-P12), and two groups were with PwMS who do not use mobility aids (P13-P24). The average age and disease duration of the participants were 50 and 16 years, respectively. Most focus group participants were female (63%), non-Hispanic White (75%), and urban area residents (96%). Descriptively, there were observed differences between user and nonuser groups in age, disease duration, and employment status (Table 3).

**Table 3:** Focus group participant characteristics.

	All (n = 24)		Mobility Aid Users (n = 12)		Nonusers (n = 12)	
Measure	*M or N*	*(SD or %)*	*M or N*	*(SD or %)*	*M or N*	*(SD or %)*
***Age***	*50.29*	*(11.0)*	55.83	*(9.1)*	44.75	*(10.1)*
***Disease Duration***	*16.02*	*(11.2)*	18.27	*(9.2)*	12.1	*(9.3)*
***Sex/Gender***						
Female	15	*(62.5%)*	8	*(66.7%)*	7	*(58.3%)*
Male	8	*(33.3%)*	4	*(33.3%)*	5	*(41.7%)*
Genderqueer/Nonbinary	1	*(4.2%)*	0	*(0.0%)*	1	*(8.3%)*
***Race and Ethnicity***						
NH White	18	*(75.0%)*	10	*(83.3%)*	8	*(66.7%)*
NH African American	5	*(20.8%)*	2	*(16.7%)*	3	*(25.0%)*
NH Mixed-race	1	*(4.2%)*	0	*(0.0%)*	1	*(8.3%)*
***Employment Status***						
Employed	10	*(41.6%)*	3	*(25.0%)*	7	*(58.3%)*
Unemployed	6	*(25.0%)*	4	*(33.3%)*	2	*(16.7%)*
Retired	7	*(29.2%)*	5	*(41.7%)*	2	*(16.7%)*
Other	1	*(4.2%)*	0	*(0.0%)*	1	*(8.3%)*
***Use a Mobility Aid***						
Use any mobility aid	12	*(50.0%)*	12	*(100%)*	/	/
Use walking aids only	3	*(12.5%)*	3	*(25%)*	/	/
Use wheeled aids	9	*(37.5%)*	9	*(75%)*	/	/
Use mixed mobility aids	4	*(16.7%)*	4	*(33%)*	/	/

### Quantitative findings

Multiple linear regression results are reported in Table 4, showing direct effects of independent variables on participation outcomes. In general, PFs (Block 1) as a group were significantly associated with all participation outcomes except with radius of gyration, with R² values ranging from 0.13 to 0.48 and p-values ranging from 0.021 to <0.001. EFs as a whole group (Block 2) had additional significant effects on satisfaction with participation (ΔR^2^ = 0.11, p = 0.002) and radius of gyration (ΔR^2^ = 0.11, p = 0.048) after accounting for PFs. When all independent variables were entered, among individual PFs, age was significantly associated with trip distance (B = 0.05, p = 0.045), physical function was significantly associated with trip frequency (B = 0.02, p = 0.021), and trip duration (B = 0.02, p = 0.030). Fatigue severity was significantly associated with perceived ability to participate (B = −0.37, p < 0.001) and satisfaction with participation (B = −0.23, p = 0.005). Among individual EFs, perceived financial resources showed significant associations with perceived ability to participate (B = 1.46, p = 0.018) and satisfaction with participation (B = 3.12, p < 0.001) after accounting for person factors in the model, while neighborhood safety had a significant association with radius of gyration (B = 1.3, p = 0.007). Perceived social support also showed a significant but negative effect on radius of gyration (B = −1.05, p = 0.022).

**Table 4:** Regression results.

Measures	Model 1: Person-related Factors				Model 2: + Environmental Factors			
	b	sr^2^	R²	p-value	b	sr^2^	ΔR²	p-value
**Outcome: Perceived Ability to Participate**								
Intercept	66.90			**<0.001**	53.90			**<0.001**
***Block 1: Personal Factors***			0.48	**<0.001**				
Age	−0.12	<0.01		0.213	−0.08	<0.01		0.384
Disease duration	0.05	<0.01		0.451	0.04	<0.01		0.482
Physical function	0.19	0.10		**<0.001**	0.12	0.02		0.050
Fatigue severity	−0.39	0.22		**<0.001**	−0.37	0.19		**<0.001**
Depression severity	−0.06	<0.01		0.265	0.02	<0.01		0.812
***Block 2: Environmental Factors***							0.05	0.079
Perceived social support					0.37	<0.01		0.653
Perceived financial resources					1.46	0.03		**0.018**
Perceived neighborhood safety					0.40	<0.01		0.644
Using walking aid only					−0.80	<0.01		0.487
Using wheeled aid					−1.70	<0.01		0.269
**Outcome: Satisfaction with Participation**								
Intercept	63.93			**<0.001**	45.87			**<0.001**
***Block 1: Personal Factors***			0.40	**<0.001**				
Age	−0.06	<0.01		0.627	−0.01	<0.01		0.915
Disease duration	0.00	<0.01		0.972	0.00	<0.01		0.981
Physical function	0.26	0.12		**<0.001**	0.14	0.02		0.057
Fatigue severity	−0.27	0.07		**0.002**	−0.23	0.05		**0.005**
Depression severity	−0.22	0.06		**0.004**	−0.11	<0.01		0.187
***Block 2: Environmental Factors***							0.11	**0.002**
Perceived social support					0.62	<0.01		0.559
Perceived financial resources					3.12	0.09		**<0.001**
Perceived neighborhood safety					−0.77	<0.01		0.485
Using walking aid only					−0.81	<0.01		0.583
Using wheeled aid					−2.18	<0.01		0.273
**Outcome: Trip Frequency**								
Intercept	0.43			0.673	0.32			0.818
***Block 1: Personal Factors***			0.13	**0.017**				
Age	0.00	<0.01		0.921	0.00	<0.01		0.921
Disease duration	0.07	<0.01		0.385	0.01	<0.01		0.403
Physical function	0.02	0.10		**0.001**	0.02	0.05		**0.021**
Fatigue severity	0.00	<0.01		0.923	−0.001	<0.01		0.943
Depression severity	−0.01	<0.01		0.424	−0.01	<0.01		0.530
***Block 2: Environmental Factors***							0.01	1.000
Perceived social support					−0.01	<0.01		0.926
Perceived financial resources					0.18	<0.01		0.844
Perceived neighborhood safety					0.01	<0.01		0.933
Using walking aid only					0.02	<0.01		0.905
Using wheeled aid					0.01	<0.01		0.958
**Outcome: Trip Distance**								
Intercept	−0.34			0.866	−2.38			0.357
***Block 1: Personal Factors***			0.13	**0.021**				
Age	0.05	0.01		0.079	0.05	0.04		**0.045**
Disease duration	−0.01	<0.01		0.505	−0.02	<0.01		0.338
Physical function	0.03	0.08		**0.005**	0.02	<0.01		0.357
Fatigue severity	0.01	<0.01		0.447	0.02	0.01		0.339
Depression severity	−0.03	0.04		0.100	−0.01	<0.01		0.530
***Block 2: Environmental Factors***							0.08	0.144
Perceived social support					−0.20	<0.01		0.378
Perceived financial resources					0.23	0.02		0.180
Perceived neighborhood safety					0.43	0.03		0.071
Using walking aid only					−0.47	0.02		0.138
Using wheeled aid					−0.59	0.02		0.162
**Outcome: Trip Duration**								
Intercept	−0.15			0.890	−0.69			0.637
***Block 1: Personal Factors***			0.14	**0.014**				
Age	0.01	<0.01		0.369	0.02	<0.01		0.303
Disease duration	0.06	<0.01		0.521	0.01	<0.01		0.617
Physical function	0.02	0.12		**<0.001**	0.02	0.04		**0.030**
Fatigue severity	0.00	<0.01		0.698	0.00	<0.01		0.788
Depression severity	−0.01	<0.01		0.568	0.00	<0.01		0.944
***Block 2: Environmental Factors***							0.03	0.744
Perceived social support					0.00	<0.01		0.997
Perceived financial resources					0.05	<0.01		0.601
Perceived neighborhood safety					0.04	<0.01		0.740
Using walking aid only					−0.24	0.02		0.178
Using wheeled aid					−0.12	<0.01		0.627
**Outcome: Radius of Gyration**								
Intercept	3.93			0.334	2.27			0.659
***Block 1: Personal Factors***			0.05	0.417				
Age	0.06	0.01		0.243	0.06	<0.01		0.227
Disease duration	−0.02	<0.01		0.600	−0.03	<0.01		0.344
Physical function	0.04	0.03		0.103	0.01	<0.01		0.734
Fatigue severity	0.02	<0.01		0.506	0.03	<0.01		0.443
Depression severity	−0.03	0.01		0.301	−0.01	<0.01		0.680
***Block 2: Environmental Factors***							0.11	**0.048**
Perceived social support					−1.05	0.05		**0.022**
Perceived financial resources					0.33	<0.01		0.324
Perceived neighborhood safety					1.30	0.07		**0.007**
Using walking aid only					−0.88	0.02		0.167
Using wheeled aid					−0.86	<0.01		0.313

*Note.* N = 100. Block 1 F-change test df = 5, 95; Block 2 df = 5, 90. Significant p-values (< 0.05) are bolded. Using wheeled aid includes both the exclusive use of wheeled aids and the combined use of wheeled aids and walking aids. Block 1 F-change test df = 5, 95; Block 2 df = 5, 90. * p < 0.05. Using wheeled aid includes both the exclusive use of wheeled aids and the combined use of wheeled aids and walking aids.

Moderation analyses were not significant, suggesting no role of mobility aid use or social support in moderating the association between PFs and participation outcomes in the sample (See Supporting Information S1 Appendix).

### Qualitative and integrated findings

A total of three overarching themes and eight subthemes emerged from focus group data. These themes are described in relation to the quantitative findings, highlighting points of convergence and divergence. The first theme elaborated on the significant associations among EFs and various aspects of community participation observed from the perspectives of participants who use or do not use mobility devices. Contrasting with the absence of effect modification of EFs shown in statistical analysis, Theme 2 illustrated the potential moderator role of environmental support in buffering the negative impact of functional impairments to improve community participation outcomes. Finally, the last theme describes strategies used by participants to navigate environmental barriers and highlights advocacy priorities for addressing systemic barriers, which were not captured in quantitative data.

### Theme 1: Environmental challenges vary by mobility aid use

While discussions focused on environmental influence, participants acknowledged how PFs (e.g., MS symptoms, personality, social roles) can affect participation outcomes, which is consistent with statistical findings. In general, qualitative findings supported the significant association between EFs and satisfaction with community participation, and provided contextual details regarding what barriers in physical, societal, and attitudinal environments influenced community participation experiences and how. Participants also implied that the magnitude of environmental influences could vary based on their mobility aid use status.

### Subtheme 1−1: Barriers in natural and built environments

Participants discussed the impact of natural environment (e.g., weather, geographical characteristics) and built environment (e.g., transportation systems, buildings) on community participation. Some participants voiced that opportunities for engaging in social activities outside the home varied based on the characteristics of one’s geographical location and surrounding community. For people who live in remote areas, interacting with peers with similar experiences outside of work can be a challenge. While acknowledging that weather can influence everyone’s ability to participate in activities outside the home, most focus group participants indicated that extreme weather conditions—such as very cold, hot, or humid weather—are demotivating due to extreme temperature or humidity intolerance, which is common among PwMS. When discussing neighborhood environment, some mobility aid users mentioned the fear of becoming an “easy target” in unsafe neighborhoods. Snow and ice, particularly, had a greater impact on mobility aid users due to unsafe sidewalks, as one shared,

*Wheelchairs don’t do well in the snow or ice. It’s difficult. And what’ll happen is if you’re on a sidewalk, the snowplows will push the snow onto the sidewalks.*

- P24, age 47, male, 12 years with MS, unemployed, mobility aid user

In addition, mobility aid users also expressed that the unavailability of public transportation, uneven surfaces on the street, and inaccessible buildings and public spaces made it difficult for them to engage in out-of-home activities as desired.

*For me, transportation is one, being in an inner city, then accessibility is hard for me to get in and out of places. Sidewalks, I think there should be a policy or something mandated to have all entries and sidewalks more wheelchair accessible because the doors that you go in now are very thin.*

- P20, age 58, male, 8 years with MS, employed, mobility aid user

Furthermore, participants provided insights on the significant statistical relationship between increased neighborhood safety with larger activity space. They explained that neighborhood safety encompassed two aspects: crime/security concerns and the physical condition of streets and sidewalks. Fear of becoming a target of attack and fall injuries could both limit their community participation.

### Subtheme 1–2: Structural gaps in support during times of vulnerability

Participants identified COVID-19, employment challenges, and difficulty in obtaining useful information as major barriers in the societal and structural environment. Participants acknowledged that while the COVID-19 pandemic impacted everyone’s life, including those without MS, they sensed additional constraints due to their immunocompromised health status and lack of others’ understanding. Between different groups of participants, those who live with invisible MS expressed stronger feelings of frustration.

*It’s not even a mindset or whatever. If I just ate in crowded bars all the time, I would get COVID all the time. I just don’t have the protection and I can’t change that… I don’t have the same level of freedom and that’s just really isolating.*

- P9, age 47, female, 7.5 years with MS, employed, nonuser

*I was immunosuppressed as well and not something that I really enjoyed, especially when they [friends] kind of diminished the fact that, “Hey guys, I’m immunosuppressed. I appreciate it if you don’t do these things and bring it back to me.” But they’re like, “Hey, we’re on vacation. We’re doing this. You knew what was happening.” I’m like, “I guess you’re right. But come on guys, be a little kind.”*

- P8, age 35, male, 12.5 years with MS, employment-other, nonuser

Work was an essential component of community participation for most participants; yet many of them shared struggles in either maintaining employment or seeking new employment after they were diagnosed. Several participants shared that they have had to resign from their jobs as their MS progressed. Some pointed out that employment challenges were often related to employers’ failure to provide reasonable job accommodation.

*They terminated me because they wouldn’t give me reasonable accommodation. And I’m like, “My doctor didn’t want me to work a 12-hour shift. He wanted me to work a 10-hour shift.” And you’re like, “Oh, well we can’t help you with that one.”*

- P4, age 39, female, 5 years with MS, employed, nonuser

Participants linked job loss to financial strain, limiting their engagement in community activities. This echoed the significant effect of perceived financial resources on subjective participation measures found in the quantitative phase. Many also noted the high cost of living with MS, due to the need for long-term healthcare services and the additional costs associated with accommodating their fatigue (e.g., taking a cab instead of walking, hiring help), with one stating she had to “budget a lot harder and cut a lot of things out” (P9, age 47, female, 7.5 years with MS, employed, nonuser).

Across groups, participants emphasized the gap between availability and accessibility of helpful resources. One commented, “There are so many things that are out there that’s able to help, but the difficulty is finding the information (P22, age 61, female, 28 years with MS, unemployed, user).” These access barriers were said to add unnecessary disease burden and emotional distress. Most mobility aid users reported few obstacles obtaining devices, however, when one participant shared that she recently got an elevating power wheelchair, P20 reflected on his past experience that demonstrated the availability-accessibility gap.

*That [trying to get an elevating power wheelchair] was a barrier for me. I remember when I first got diagnosed with MS, I tried to get that elevator chair and they did everything they could possibly do for me not to get that seat. I don’t understand why they make something for people for convenience, especially those that have a disability, but make it so hard for you to get it, especially if you’re a minority.*

- P20, age 58, male, 8 years with MS, employed, mobility aid user

### Subtheme 1–3: Barriers in the attitudinal environment

Participants expressed that the attitudinal environment—formed by the attitudes of others, including acquaintances and strangers—influenced their motivation and satisfaction with community participation. Several participants across groups implied that ableist attitudes exist in the general public. They have encountered or witnessed negative attitudes from strangers who showed little care for people with disabilities. For example, a participant shared that she once received a note with hateful words on her car that was parked at an accessible spot in a school. Others reflected on how witnessing ableism has transformed into internalized stigma.

*I saw the treatment of my mom. She was in a wheelchair. I have three memories of her walking, ever. And she got the stigma of, “Oh, she can’t do this, she can’t do that, and we’re going to look at her funny and we’re going to ask other people about it and talk about her behind her back.” And that’s a stigma that I have from childhood… And now my husband has the excitement of me in a wheelchair, and my Dad’s like, “Oh, you married her. She’s your problem now.” And after Dad dealt with Mom for so many years, you are stuck in this mental space of, “Is it on me? Is it my perception of reality? What is it?”*

- P23, age 44, female, 5 years with MS, employed, mobility aid user

Discussions around disclosing MS only came up in nonuser groups. They stressed that whether to disclose their diagnosis is a personal choice, but if they chose to not disclose, it was often due to fear of stigma. Specifically, they expressed a strong denial of disclosing MS at workplaces unless necessary when requesting job accommodation because they do not want to be labeled as “disabled” and “incompetent.”

*I had a somewhat obnoxious diagnostic path where doctor didn’t believe me. When they finally diagnosed me, he was pretty flippant and they just kind of thrust a brochure at me about MS. And in my memory, emblazoned on this brochure was basically, “Be careful who you tell, people may be prejudiced against you.” …I felt extremely isolated all of a sudden and that I was no longer in control of my own destiny, that people would judge me and predefine and assume what I was capable of with the diagnosis if I shared it… For better or worse, I guess we do live in a society where people, especially in employment opportunities, can be prejudged based on what they bring to the table in terms of physical and mental ability.*

- P11, age 33, female, 10 years with MS, employed, nonuser

### Theme 2: Supportive environments ease the burden of MS symptoms

This theme describes participant perspectives about external supports promoting their out-of-home participation despite the co-existing negative impact of MS-related impairments. Environmental supports included accessible services and resources, support networks, and the use of mobility aids. While quantitative results showed that the use of wheeled aids was significantly correlated with worse community participation, focus group findings indicate that using mobility aids re-enabled and facilitated community participation. In addition, focus group findings illustrated how social support and the use of mobility aids, especially wheeled aids, might mitigate the negative impact of MS-related symptoms on community participation, contrasting the insignificance found in the quantitative phase.

### Subtheme 2−1: Having access to resources and opportunities

Having access to community resources and opportunities to engage as active members was seen as a facilitator. Two participants were involved in university-led disability education projects, and they viewed this opportunity as affirming and motivating to engage in activities outside the home. P24 (age 47, male, 12 years with MS, unemployed, mobility aid user), who lost the ability to drive a standard vehicle due to impaired lower limb mobility, credited vehicle modifications and adaptive driving training for enabling continued community access. In addition, several participants complimented disability services, such as priority boarding at airports, accessible gyms, and accessible public transportation, for supporting people with long-term disabilities in remaining engaged in the community.

*…When it got to the point where I couldn’t work, and I couldn’t drive, I was by myself a lot... The para-transit shuttle, which picked me up where I lived, took me wherever I was going. Because of that, I started to get involved with things again, I got involved in things for people with disabilities, and started going to an MS support group.*

- P18, age 62, female, 22 years with MS, retired, user

### Subtheme 2−2: Social support from a close network

Contrary to regression results suggesting a negative association between social support and radius of gyration or activity space, focus group participants considered having a caring and understanding support network as positive and important. Many reported a shrinking social network shortly post-diagnosis, especially among distant acquaintances, but expressed gratitude for those who remained—close friends, colleagues, and family members. For example, P8 (age 35, male, 12.5 years with MS, employment-other, nonuser) appreciated his co-workers’ understanding when he had to be absent during relapses. Another participant whose work environment was less supportive described her small but strong circle of close friends as her main support.

*After coming to terms with what it meant for me to be living with MS, I started to share with a couple of close friends, and people were so supportive, and kind, and loving, and we’ve established a history of doing the Walk MS and also Bike MS in [location]. This will be our seventh year doing it.*

- P11, age 33, female, 10 years with MS, employed, nonuser

In addition to personal networks, many participants noted the growing availability of community resources specific to the MS population in recent years. They praised the National MS Society for serving as a central hub of information. Many described MS support groups as a key source for social connection, where they found a sense of community and learned strategies from peers.

### Subtheme 2–3: Use of mobility aids

All mobility aid users shared that the devices enabled them to travel and engage in desired activities, which would otherwise be restricted by their mobility impairments. However, many admitted that when they first found out they had to rely on a device, they struggled to accept it. As P15 (age 42, female, 11 years with MS, unemployed, user) stated, “*I went through that [stigma phase]. I was so embarrassed to use it*,” mobility devices were often perceived as a symbol of “disabled” and “incapable.” Many resonated with that experience and shared that disability stigma takes time to overcome. They described that, however, over time, they recognized mobility aids as empowering.

*I have a walker, I have a wheelchair, I have canes. It just depends on how my fatigue level is that day…. It’s [using mobility aid devices is] helping me stay involved, it’s helping me to still get out there and participate and just making myself happy. (P22)*

### Theme 3: Individual efforts are essential, but more needs to be done at the systemic level

This theme provides further understanding of how people navigate environmental barriers and what needs to be improved in the systemic environment, which was not captured by the quantitative data. Participants highlighted that although acceptance, optimism, and adaptation are helpful strategies, greater collective efforts in society to support PwMS are warranted.

### Subtheme 3−1: Acceptance, optimism, and adaptation are helpful strategies

Most participants who have lived with MS for a long time shared that, over the years, they have developed acceptance of MS and their limitations, which has enabled them to approach barriers with a positive mindset. One stated, “*That was very hard to learn at first, to accept, and ask for that help. But now I know that the only way I can do things is I need that help”* (P17, age 66, female, 33 years with MS, retired, user). Optimism was viewed as an empowering skill for reducing distress, as one advised, “*Don’t concentrate on what you can’t do. Concentrate on what you can do, concentrate on your strengths*” P14 (age 58, male, 24-year diagnosis, unemployed, user).

Another helpful strategy was proactively assessing needs and adapting behaviors when changing the environment is not possible. Over time, participants have learned what worked to stay engaged, as P17 (age 66, female, 33 years with MS, retired, user) stated, “*I’ve lived with MS for so long, and I’m in the power wheelchair, I know what it’s going to take to accomplish something*.” This ability was described as not simply innate, but rather a learning process shaped by experiences. Recognizing the fluctuating nature of MS, some participants have learned to be flexible in planning and pacing activities based on the energy level and priority of tasks. For example, P11 (age 33, female, 10 years with MS, employed, nonuser), who loves to jog outdoors, would switch to biking in summer to manage heat intolerance. Lastly, participants described that actively seeking information and resources, such as looking for accessible restaurants, planning an accessible route, and asking for help, is part of their proactive adaptation.

### Subtheme 3−2: Advocacy for enhancing environmental supports

Although participants were able to achieve some level of success in optimizing community engagement through extensive individual efforts, they advocated for increasing systemic support. Across focus groups, four advocacy priorities were identified: Improving (1) built environment accessibility, (2) ease of access to information, (3) MS specialty care, and (4) public attitudes toward disability. Participants noted that the existing Americans with Disabilities Act (ADA) law mandates an accessible built environment, but the problem is around the lack of enforcement and quality monitoring.

*Most of the world isn’t ADA accessible. So, being active, there’s lots of barriers to getting in and out of places. Even when they say they’re ADA accessible, they’re full of shit.*

- P18, age 62, female, 22 years with MS, retired, user

*A lot of this issue in my opinion boils down to your local municipalities... You get the handout or the annual update that says, “You homeowners have to shovel your sidewalks,” but no one, no one, enforces it. So that’s where we’re stuck.*

- P21, age 65, female, 10 years with MS, retired, user

Reflecting on their own experiences, participants stressed the need to streamline access to information, especially for those recently diagnosed, who often feel overwhelmed and unaware of available resources. As P21 (age 65, female, 10 years with MS, retired, user) stated, “*they’re in shell shock. It’s so devastating at the time. You don’t know what’s going to happen next*.” Therefore, increasing the awareness of existing resources is key.

Improving MS specialty care was another priority, with calls for expanding access and coverage to MS-specialized clinics nationwide and increasing the number of providers with MS expertise. A few participants pointed out the lack of service for MS in their regions. One participant shared, “*Where I live here in [location name] we just had built a multimillion-dollar hospital … there is no definite support group advocate in the [location name] area for the MS Society… I’m really hoping that we’re going to get more proactive with the medicines in this area because of this hospital*” (P18, age 62, female, 22 years with MS, retired, user). Several participants shared dissatisfactory interactions with healthcare providers related to delayed diagnosis, not feeling trusted, and receiving poor advice regarding exercise.

Lastly, participants urged changes in public attitudes toward disabilities. Having experienced misunderstanding and discrimination, they emphasized the need for everyone to be more caring, understanding, and empathetic, especially toward those with invisible disabilities. Many mobility aid users expressed that most people seemed respectful, and some acted even more kindly after they noticed their mobility devices. However, those with invisible MS shared a different perspective, as one put it, “*As a society, we have a hard time putting empathy into those things that we cannot see*” (P1, age 55, female, 29 years with MS, retired, nonuser). Therefore, addressing negative attitudes is particularly essential for those with invisible disabilities.

## Discussion

This study explored how physical, societal, and attitudinal environments influence community participation outcomes and identified the systemic support needed to enhance community participation from the perspectives of PwMS. Regression results revealed that PFs as a group significantly explained variance in most community participation outcomes. When PFs were accounted for, EFs explained an additional variance in both satisfaction with participation and radius of gyration. There was no evidence of statistically significant moderation by EFs of hypothesized PF-participation relationships. In comparison, although focus group participants noted the negative impact of PFs, they explained why and how EFs matter in supporting community participation, especially when functioning declines. They also emphasized how individual efforts are limited in navigating barriers without system-level support. Together, these mixed methods findings deepened our understanding of EFs’ role in shaping community participation among PwMS and provided implications for practice and future research.

Among individual PFs, physical function and fatigue showed significant associations with some participation outcomes, consistent with prior studies and the focus of rehabilitation strategies on symptom self-management and physical activities [14,21,53]. Among individual EFs analyzed, perceived financial resources demonstrated the strongest association with subjective participation perspectives, while perceived neighborhood safety is independently associated with greater radius of gyration. Focus group data echoed these associations, noting that financial stress and unsafe environments discourage out-of-home participation. These results are supported by Hall et al. [54], who found lower income associated with decreased social participation, and Desai et al. [55], whose scoping review highlighted the role of neighborhood safety in affecting activity space among adults with physical disabilities. Interestingly, perceived social support showed a negative association with radius of gyration, contrary to focus group data and prior findings [56]. One possible explanation is that PwMS with stronger support networks may rely more on close, local connections [57], resulting in smaller activity spaces despite high perceived engagement. Social support is multidimensional, including emotional, instrumental, and informational support, and can be influenced by the size and density of the social network [58]. It is possible that different aspects of social support have various associations with different aspects of participation, but our quantitative data did not capture all aspects of social support. We should also acknowledge that a larger activity space itself does not equate with meaningful participation. Further research should explore how social support affects spatial aspects of participation. For example, Geographically-explicit Ecological Momentary Assessment (GEMA) could provide granular data on the characteristics of specific locations and times of activities that involve participation [59].

Our quantitative findings indicated that symptom severity had a greater influence on participation than EFs. However, as Noreau and Boschen [60] noted, the lack of significant statistical findings often does not conclude an absence of environmental influence on community participation in the real world. In reality, environmental influences are often underestimated by empirical statistics due to the difficulty in quantifying contextual nuances. In our study, the weak association could be that only a few self-reported EF variables were analyzed due to the limited availability in the existing dataset. Other significant EFs, such as access to public transportation and the objective measures of built environment—reported in previous literature [55,61] and highlighted by the focus group participants—were not measured. It is also possible that in our sample, many people reported higher environmental support despite facing increased participation challenges because of optimism, resulting in limited statistical evidence for a positive relationship between supportive EFs and participation outcomes [62]. In our study, qualitative data offered critical context and insights into how perceived EFs hinder or facilitate community participation experiences.

In addition to supporting the statistical significance of environmental influences, qualitative findings identified major environmental barriers to community participation: unfavorable location, weather, inaccessible built environment, COVID-19, employment challenges, difficulty obtaining useful information, internalized stigma, and ableist attitudes. These aligned with barriers reported by people with disabilities and stroke survivors [63,64]. Interestingly, participants using mobility devices reported more barriers in the built environment, whereas those living with invisible MS emphasized fear of MS disclosure and concerns about attitudinal environments. The invisibility of MS often leads to others’ lack of understanding or disbelief in PwMS’ struggles, resulting in a feeling of isolation [3]. Notably, although qualitative data were collected in 2024, participants described lasting impacts of the COVID-19 pandemic on community participation. These findings are consistent with Morris-Bankole and Ho [65], who reported that COVID-19 posed substantial challenges for many immunocompromised PwMS, and with findings that COVID-19 has widened participation disparities between people with and without disabilities [66]. Future studies should investigate the pandemic’s long-term effects on participation among PwMS.

This study offered valuable insights into the role of mobility aid use. Our regression results did not show a significant link between using mobility aids and participation outcomes. In fact, it is not uncommon for wheelchair use to be identified as a risk factor for reduced participation statistically [67]. However, focus group participants viewed mobility aids as re-enabling, emphasizing their role in restoring independence and access to valued community activities. Taken together, our findings suggest a complex pathway linking physical impairment, mobility aid use, and participation outcomes. The absence of statistical evidence for a positive effect of mobility aid use may reflect the mediating role of physical impairment: declining physical function can directly limit participation while simultaneously increasing reliance on mobility aids. In this context, mobility aid use may have a re-enabling effect, but its positive influence on participation can be obscured in statistical models by the stronger, direct negative effects of physical impairment. Whether mobility aid use ultimately supports participation also likely depends on environmental support and personal adaptation. As discussed by Widehammar et al. [68], while mobility devices are designed to facilitate community engagement, various barriers can diminish their supportive function. These barriers include inaccessible environment, lack of training, lack of social support, internalized stigma, and unfavorable weather [69]. Therefore, unless all confounding contextualized and personal factors are accounted for, the potential of mobility aids to enhance participation may remain obscured in statistical models.

The study identified priorities for increasing system-level support to promote community participation for PwMS. While participants described persistent efforts to navigate environmental barriers, they stressed that individual action alone is insufficient without broader systemic intervention. This highlights the continued need for advocacy and collective actions, alongside advances in symptom-level MS treatments such as disease-modifying treatments, restorative rehabilitation therapies, and symptom self-management interventions. Rehabilitation researchers have long called for system-level environmental supports to enable participation, as Whiteneck and Dijkers wrote, “*Modern medicine can still not eliminate many activity limitations, but at least in theory, with the right assistive devices, personal assistance, social support, policies, and environment, people with disability can fully participate in society* [70, p. S24].” Advocacy topics raised by study participants were MS-specific but aligned with broader disability community priorities [61,71,72]. Future research should seek actionable strategies to advance these goals.

Several limitations should be noted. First, this study was exploratory in nature, and the number of statistical tests conducted may increase risk of family-wise error. Second, although the study is one of only a few studies utilizing GPS data in MS (e.g., Neven et al. [73]) and represents the largest sample size published in this population using the methodology, our sample size remained relatively small, which limits stability and generalizability. Furthermore, the included EFs were self-reported data, which may explain stronger associations with subjective participation outcomes compared to the GPS-measured variables. Different results may be expected with objectively measured EFs derived from a combination of GPS geographic information system (GIS) data, as suggested by Chan et al. [36] and Magasi et al. [74].

We also acknowledge that only three pairs of EF-PF interactions were examined statistically, and other possible permutations were not assessed. Future studies may consider using forward selection or other data-driven screening methods to build regression models. Discrepancies between the quantitative and qualitative data should be noted when interpreting the findings. While the quantitative data were collected from 2019−2021, focus groups were held in early 2024. The COVID-19 pandemic may have influenced community participation outcomes among quantitative phase participants. Additionally, while the study focused on out-of-home participation, PROMIS-AP and PROMIS-SRA inquire about activities that could occur both at home and outside the home (e.g., leisure activities, family activities, work).

Despite these limitations, this study makes a unique contribution to the existing MS literature and participation research by adopting a mixed-methods design, integrating patient-reported outcomes, GPS-derived measures, and lived experiences. Importantly, our findings highlight how environmental influences may operate in complex and context-dependent ways that are not always captured by quantitative models, demonstrating the value of mixed-methods inquiry. The study also identified potentially helpful solutions to support community participation for PwMS at individual and systemic levels. Further studies should build on these findings to develop, test, and optimize interventions and policies that empower PwMS to navigate both internal and contextual barriers to participation and promote environments that facilitate community participation.

## Supporting information

S1 Appendix Results of moderation analysis. (DOCX)

S2 Dataset Deidentified dataset. (XLSX)
