# Supplementary material for: Environmental influences on community participation among people with multiple sclerosis: A mixed methods study
Source: PLoS One. 2026 Feb 10;21(2):e0342678. doi: 10.1371/journal.pone.0342678 (PMC12970384; doi:10.1371/journal.pone.0342678)
Supplement: S1 Appendix — (DOCX) [file pone.0342678.s001.docx]

**S1 Appendix. Results of Moderation Analysis**

| **Table S1.** Moderation of the Use of Mobility Aids on the Association between Physical Function and Community Participation | | | | | | | |
| --- | --- | --- | --- | --- | --- | --- | --- |
| **Measures** | **Model 1: Demographics, Physical Function, and Mobility Aids** | | |  | **Model 2: + Effect Modification** | | |
|  | **B** | **sr^2^** | **R²** |  | **b** | **sr^2^** | **ΔR²** |
|  |  |  |  |  |  |  |  |
| **Outcome: Perceived ability to participate** | | | | | | | |
| Intercept | 33.96* |  |  |  | 27.91* |  |  |
| ***Block 1*** |  |  | 0.16* |  |  |  |  |
| Age | -0.01 | <0.01 |  |  | 0.01 | <0.01 |  |
| Disease duration | 0.08 | <0.01 |  |  | 0.07 | <0.01 |  |
| Physical functions | 0.20* | 0.07 |  |  | 0.30 | 0.03 |  |
| Using walking aid only | -1.39 | <0.01 |  |  | 1.53 | <0.01 |  |
| Using wheeled aid | -0.77 | <0.01 |  |  | 6.42 | <0.01 |  |
| ***Block 2*** |  |  |  |  |  |  | 0.01 |
| Using walking aid only X PF | |  |  |  | -0.05 | <0.01 |  |
| Using wheeled aid X PF |  |  |  |  | -0.17 | <0.01 |  |
|  |  |  |  |  |  |  |  |
| **Outcome: Satisfaction with participation** | | | | | | | |
| Intercept | 30.09* |  |  |  | 39.07* |  |  |
| ***Block 1*** |  |  | 0.19* |  |  |  |  |
| Age | 0.06 | <0.01 |  |  | 0.04 | <0.01 |  |
| Disease duration | 0.02 | <0.01 |  |  | 0.04 | <0.01 |  |
| Physical functions | 0.26* | 0.07 |  |  | 0.10 | <0.01 |  |
| Using walking aid only | -1.90 | <0.01 |  |  | -15.59 | 0.01 |  |
| Using wheeled aid | -1.56 | <0.01 |  |  | -9.04 | <0.01 |  |
| ***Block 2*** |  |  |  |  |  |  | 0.01 |
| Using walking aid only X PF | |  |  |  | 0.30 | 0.01 |  |
| Using wheeled aid X PF |  |  |  |  | 0.14 | <0.01 |  |
|  |  |  |  |  |  |  |  |
| **Outcome: Trip frequency** | | | | | | | |
| Intercept | -0.10 |  |  |  | -1.41 |  |  |
| ***Block 1*** |  |  | 0.13* |  |  |  |  |
| Age | 0.00 | <0.01 |  |  | 0.00 | <0.01 |  |
| Disease duration | 0.01 | 0.07 |  |  | 0.01 | <0.01 |  |
| Physical functions | 0.02* | 0.06 |  |  | 0.05* | 0.05 |  |
| Using walking aid only | 0.01 | <0.01 |  |  | 1.15 | <0.01 |  |
| Using wheeled aid | 0.03 | <0.01 |  |  | 1.42 | 0.01 |  |
| ***Block 2*** |  |  |  |  | -0.02 |  | 0.02 |
| Using walking aid only X PF | |  |  |  | -0.03 | <0.01 |  |
| Using wheeled aid X PF |  |  |  |  | -0.03 | 0.02 |  |
|  |  |  |  |  |  |  |  |
| **Outcome: Trip distance** | | | | | | | |
| Intercept | -0.61 |  |  |  | -0.88 |  |  |
| ***Block 1*** |  |  | 0.13* |  |  |  |  |
| Age | 0.05 | 0.04 |  |  | 0.05 | 0.04 |  |
| Disease duration | -0.01 | <0.01 |  |  | -0.01 | <0.01 |  |
| Physical functions | 0.03 | 0.03 |  |  | 0.03 | <0.01 |  |
| Using walking aid only | -0.49 | 0.02 |  |  | 0.04 | <0.01 |  |
| Using wheeled aid | -0.38 | <0.01 |  |  | -0.20 | <0.01 |  |
| ***Block 2*** |  |  |  |  |  |  | 0.00 |
| Using walking aid only X PF | |  |  |  | -0.01 | <0.01 |  |
| Using wheeled aid X PF |  |  |  |  | 0.00 | <0.01 |  |
|  |  |  |  |  |  |  |  |
| **Outcome: Trip duration** | | | | | | | |
| Intercept | -0.63 |  |  |  | -0.58 |  |  |
| ***Block 1*** |  |  | 0.15* |  |  |  |  |
| Age | 0.16 | <0.01 |  |  | 0.02 | 0.01 |  |
| Disease duration | 0.01 | 0.01 |  |  | 0.01 | <0.01 |  |
| Physical functions | 0.02* | 0.06 |  |  | 0.02 | 0.01 |  |
| Using walking aid only | -0.25 | 0.02 |  |  | -0.31 | <0.01 |  |
| Using wheeled aid | -0.08 | <0.01 |  |  | -0.12 | <0.01 |  |
| ***Block 2*** |  |  |  |  |  |  | 0.00 |
| Using walking aid only X PF | |  |  |  | 0.00 | <0.01 |  |
| Using wheeled aid X PF |  |  |  |  | 0.00 | <0.01 |  |
|  |  |  |  |  |  |  |  |
| **Outcome: Radius of gyration** | | | | | | | |
| Intercept | 3.99 |  |  |  | 3.98 |  |  |
| ***Block 1*** |  |  | 0.06 |  |  |  |  |
| Age | 0.07 | 0.02 |  |  | 0.07 | 0.02 |  |
| Disease duration | -0.02 | <0.01 |  |  | -0.02 | <0.01 |  |
| Physical functions | 0.04 | 0.01 |  |  | 0.03 | <0.01 |  |
| Using walking aid only | -0.81 | 0.02 |  |  | -2.20 | <0.01 |  |
| Using wheeled aid | -0.37 | <0.01 |  |  | 0.10 | <0.01 |  |
| ***Block 2*** |  |  |  |  |  |  | 0.05 |
| Using walking aid only X PF | |  |  |  | 0.33 | <0.01 |  |
| Using wheeled aid X PF |  |  |  |  | -0.02 | <0.01 |  |
| *Note.* N=100. * indicates p < 0.05. Using walking aid only X PF = Interaction between using walking aids only and physical functions; Using wheeled aid X PF = Interaction between using wheeled aids and physical function. Using wheeled aid includes both the exclusive use of wheeled aids and the combined use of wheeled aids and walking aids. A natural log transformation method was applied to three outcome variables: trip distance, trip duration, and radius of gyration. | | | | | | | |

| **Table S2.** Moderation of Social Support on the Association between Fatigue Severity and Community Participation | | | | | | | |
| --- | --- | --- | --- | --- | --- | --- | --- |
| **Measures** | **Model 1: Demographics, Social Support, and Fatigue Severity** | | |  | **Model 2: + Effect Modification** | | |
|  | **b** | **sr^2^** | **R²** |  | **b** | **sr^2^** | **ΔR²** |
|  |  |  |  |  |  |  |  |
| **Outcome: Perceived ability to participate** | | | | | | | |
| Intercept | 71.45* |  |  |  | 85.12* |  |  |
| ***Block 1*** |  |  | 0.39* |  |  |  |  |
| Age | -0.21* | 0.03 |  |  | -0.21* | 0.03 |  |
| Disease duration | 0.03 | <0.01 |  |  | 0.04 | <0.01 |  |
| Fatigue severity | -0.41* | 0.29 |  |  | -0.62 | 0.02 |  |
| Social support | 1.36 | 0.02 |  |  | -1.61 | <0.01 |  |
| ***Block 2*** |  |  |  |  |  |  | 0.00 |
| Social support X Fatigue |  |  |  |  | 0.05 | <0.01 |  |
|  |  |  |  |  |  |  |  |
| **Outcome: Satisfaction with participation** | | | | | | | |
| Intercept | 64.16* |  |  |  | 44.78 |  |  |
| ***Block 1*** |  |  | 0.24* |  |  |  |  |
| Age | -0.17 | 0.01 |  |  | -0.17 | 0.01 |  |
| Disease duration | -0.02 | <0.01 |  |  | -0.03 | <0.01 |  |
| Fatigue severity | -0.36* | 0.14 |  |  | -0.06 | <0.01 |  |
| Social support | 2.29* | 0.04 |  |  | 6.49 | <0.01 |  |
| ***Block 2*** |  |  |  |  |  |  | 0.00 |
| Social support X Fatigue |  |  |  |  | -0.07 | <0.01 |  |
|  |  |  |  |  |  |  |  |
| **Outcome: Trip frequency** | | | | | | | |
| Intercept | 1.64 |  |  |  | 3.45 |  |  |
| ***Block 1*** |  |  | 0.02 |  |  |  |  |
| Age | -0.01 | <0.01 |  |  | -0.01 | 0.01 |  |
| Disease duration | 0.01 | <0.01 |  |  | 0.01 | <0.01 |  |
| Fatigue severity | -0.01 | <0.01 |  |  | -0.03 | <0.01 |  |
| Social support | 0.04 | <0.01 |  |  | -0.35 | <0.01 |  |
| ***Block 2*** |  |  |  |  |  |  | 0.004 |
| Social support X Fatigue |  |  |  |  | 0.01 | <0.01 |  |
|  |  |  |  |  |  |  |  |
| **Outcome: Trip distance** | | | | | | | |
| Intercept | 0.36 |  |  |  | -0.39 |  |  |
| ***Block 1*** |  |  | 0.03 |  |  |  |  |
| Age | 0.03 | <0.01 |  |  | 0.03 | <0.01 |  |
| Disease duration | -0.01 | <0.01 |  |  | -0.01 | <0.01 |  |
| Fatigue severity | 0.00 | <0.01 |  |  | 0.01 | <0.01 |  |
| Social support | 0.22 | 0.04 |  |  | 0.38 | <0.01 |  |
| ***Block 2*** |  |  |  |  |  |  | 0 |
| Social support X Fatigue |  |  |  |  | 0.00 | <0.01 |  |
|  |  |  |  |  |  |  |  |
| **Outcome: Trip duration** | | | | | | | |
| Intercept | 0.86 |  |  |  | 2.52 |  |  |
| ***Block 1*** |  |  | 0.04 |  |  |  |  |
| Age | 0.00 | 0.01 |  |  | 0.00 | 0.01 |  |
| Disease duration | 0.01 | <0.01 |  |  | 0.01 | <0.01 |  |
| Fatigue severity | -0.01 | <0.01 |  |  | -0.03 | <0.01 |  |
| Social support | 0.13 | 0.01 |  |  | -0.22 | <0.01 |  |
| ***Block 2*** |  |  |  |  |  |  | 0 |
| Social support X Fatigue |  |  |  |  | 0.01 | <0.01 |  |
|  |  |  |  |  |  |  |  |
| **Outcome: Radius of gyration** | | | | | | | |
| Intercept | 1.09 |  |  |  | 2.20 |  |  |
| ***Block 1*** |  |  | 0.02 |  |  |  |  |
| Age | 0.00 | <0.01 |  |  | 0.00 | <0.01 |  |
| Disease duration | 0.00 | <0.01 |  |  | 0.01 | <0.01 |  |
| Fatigue severity | -0.01 | <0.01 |  |  | -0.02 | <0.01 |  |
| Social support | 0.08 | <0.01 |  |  | -0.16 | <0.01 |  |
| ***Block 2*** |  |  |  |  |  |  | 0.001 |
| Social support X Fatigue |  |  |  |  | 0.00 | <0.01 |  |
| *Note.* N=100. * indicates p < 0.05. Social support X Fatigue = Interaction between perceived social support and fatigue severity. A natural log transformation method was applied to three outcome variables: trip distance, trip duration, and radius of gyration. | | | | | | | |
|  |  |  |  |  |  |  |  |
|  |  |  |  |  |  |  |  |
| **Table S3.** Moderation of Social Support on the Association between Depression Severity and Community Participation | | | | | | | |
| **Measures** | **Model 1: Demographics, Social Support, and Depression Severity** | | |  | **Model 2: + Effect Modification** | | |
|  | **b** | **sr^2^** | **R²** |  | **b** | **sr^2^** | **ΔR²** |
|  |  |  |  |  |  |  |  |
| **Outcome: Perceived ability to participate** | | | | | | | |
| Intercept | 54.33* |  |  |  | 75.52* |  |  |
| ***Block 1*** |  |  | 0.18* |  |  |  |  |
| Age | -0.16 | 0.01 |  |  | -0.16 | 0.01 |  |
| Disease duration | 0.07 | <0.01 |  |  | 0.08 | <0.01 |  |
| Depression severity | -0.21* | 0.08 |  |  | -0.57 | 0.02 |  |
| Social support | 1.34 | 0.02 |  |  | -3.16 | <0.01 |  |
| ***Block 2*** |  |  |  |  |  |  | 0.01 |
| Social support X depression | |  |  |  | 0.08 | <0.01 |  |
|  |  |  |  |  |  |  |  |
| **Outcome: Satisfaction with participation** | | | | | | | |
| Intercept | 62.12* |  |  |  | 64.35* |  |  |
| ***Block 1*** |  |  | 0.23* |  |  |  |  |
| Age | -0.16 | 0.01 |  |  | -0.16 | 0.01 |  |
| Disease duration | 0.00 | <0.01 |  |  | 0.01 | <0.01 |  |
| Depression severity | -0.33* | 0.13 |  |  | -0.37 | <0.01 |  |
| Social support | 1.51 | 0.02 |  |  | 1.04 | <0.01 |  |
| ***Block 2*** |  |  |  |  |  |  | 0.00 |
| Social support X depression | |  |  |  | 0.01 | <0.01 |  |
|  |  |  |  |  |  |  |  |
| **Outcome: Trip frequency** | | | | | | | |
| Intercept | 2.11 |  |  |  | 3.51 |  |  |
| ***Block 1*** |  |  | 0.03 |  |  |  |  |
| Age | -0.01 | 0.01 |  |  | -0.01 | 0.01 |  |
| Disease duration | 0.01 | <0.01 |  |  | 0.01 | <0.01 |  |
| Depression severity | -0.01 | 0.01 |  |  | -0.03 | <0.01 |  |
| Social support | 0.00 | <0.01 |  |  | -0.29 | <0.01 |  |
| ***Block 2*** |  |  |  |  |  |  | 0.003 |
| Social support X depression | |  |  |  | 0.01 | <0.01 |  |
|  |  |  |  |  |  |  |  |
| **Outcome: Trip distance** | | | | | | | |
| Intercept | 2.54 |  |  |  | 6.90 |  |  |
| ***Block 1*** |  |  | 0.05 |  |  |  |  |
| Age | 0.02 | <0.01 |  |  | 0.02 | <0.01 |  |
| Disease duration | -0.01 | <0.01 |  |  | -0.01 | <0.01 |  |
| Depression severity | -0.02 | 0.02 |  |  | -0.10 | 0.01 |  |
| Social support | 0.10 | <0.01 |  |  | -0.98 | <0.01 |  |
| ***Block 2*** |  |  |  |  |  |  | 0.01 |
| Social support X depression | |  |  |  | 0.02 | 0.01 |  |
|  |  |  |  |  |  |  |  |
| **Outcome: Trip duration** | | | | | | | |
| Intercept | 1.31 |  |  |  | 1.79 |  |  |
| ***Block 1*** |  |  | 0.02 |  |  |  |  |
| Age | 0.00 | <0.01 |  |  | -0.01 | <0.01 |  |
| Disease duration | 0.01 | <0.01 |  |  | 0.01 | <0.01 |  |
| Depression severity | -0.01 | 0.01 |  |  | -0.02 | <0.01 |  |
| Social support | 0.05 | <0.01 |  |  | -0.06 | <0.01 |  |
| ***Block 2*** |  |  |  |  |  |  | 0 |
| Social support X depression | |  |  |  | 0.00 | <0.01 |  |
|  |  |  |  |  |  |  |  |
| **Outcome: Radius of gyration** | | | | | | | |
| Intercept | 10.33* |  |  |  | 16.13 |  |  |
| ***Block 1*** |  |  | 0.02 |  |  |  |  |
| Age | 0.03 | <0.01 |  |  | 0.03 | <0.01 |  |
| Disease duration | -0.02 | <0.01 |  |  | -0.02 | <0.01 |  |
| Depression severity | -0.04 | 0.02 |  |  | -0.14 | 0.01 |  |
| Social support | -0.25 | <0.01 |  |  | -1.48 | <0.01 |  |
| ***Block 2*** |  |  |  |  |  |  | 0.003 |
| Social support X depression |  |  |  |  | 0.02 | <0.01 |  |
| *Note. N=100. * indicates p < 0.05. Social support X depression = Interaction between perceived social support and depression severity. A natural log transformation method was applied to three outcome variables: trip distance, trip duration, and radius of gyration.* | | | | | | | |
